# On a Particular Class of Dreams Induced by Food

**Published:** 1858-10-01

**Authors:** 


### ART. IV.—ON A PARTICULAR CLASS OF DREAMS INDUCED BY FOOD.

> "And Phan'sie, I tell you, has dreams that have wings,
> And dreams that have honey, and dreams that have stings,
> Dreams of the maker, and dreams of the teller,
> *Dreams of the kitchen, and dreams of the cellar.*"—BEN JONSON.

IN two previous Essays "On Certain Physiological and Psychological Phenomena of Dreams" in this Journal, we endeavoured to establish the opinions that these different "visions of the night" were induced by certain conditions of the brain; by partial states of vigilance of the external senses; by the agency of certain physical influences, such as heat, cold, light, and so forth; by pressure on the heart and the other organs of circulation; and by particular conditions of the muscular system.

We now propose to investigate several interesting phenomena arising from the effects of different kinds of food, particularly when taken for supper, in order to show that special trains of thought are induced by what is taken at this unwholesome meal. The investigation will have a psychological bearing, by showing that all deranging influences or disturbing tendencies affect the brain. Yet the combination and accretion of new ideas which are thus engendered, and the extraordinary powers which the mind seems to possess under such circumstances over time and space, furnish matter for profound reflection on the independence of the soul when it induces the complexities of any special vision; which phenomena furnish presumptive proof of the soul's actual existence, and that it will still exist when the mortal coil is thrown off altogether.

Instead, therefore, of urging, as some have done, that, if so many extraneous influences may be the cause of dreaming, by inducing trains of thought which for the time occupy the dreamer's consciousness, that these phenomena lead to the inevitable corollary of the materiality of mind, and also to the conclusion that these trains of ideas are merely acts of cerebration, such inferences seem to us not only gratuitous, but also highly unphilosophical. All that these phenomena actually show is this, that everything which affects the circulation of the blood, or which increases or diminishes the *vis nervosa*, may, and does interfere, modify, or stimulate the action of the mental faculties; so far as any material agent can affect the condition of the organs of the mind, by exalting or diminishing their respective functions.

The effects on the cerebral organization are precisely similar to other organic conditions; as, for instance, when the eye-balls suffer from any inflammatory action, vision for the time will be defective. And we might state as a physiological proposition, when speaking of this condition, that, although the soul is temporarily deprived of the use of the optical instruments for communicating with the outer world, yet this deprivation does not interfere with its absolute convictions that all surrounding objects continue to possess proportion, consistency, form and colour, even though it cannot recognise them.

We may therefore assume that if, when dreaming, we have the power of painting scenes marvellously striking or exquisitely beautiful, these cannot be regarded as induced by organic impressions, but by the inherent power of the soul, which dimly perceives its own vast capacity and sublime destiny. And, although the immortal mind may seemingly be affected by a fever, or disturbed by a debauch, inducing particular dreaming tendencies, yet the very visions are so different from matter-of-fact occurrences, that, instead of furnishing arguments damaging the sublime and independent functions of the soul, it seems to us to render this independence rather more a matter of certainty than as being anything of a speculative character.

In a word, we regard the fact as established, that dreams merely indicate certain disturbed conditions of the organic instruments of the mental faculties, through which the soul manifests its power and capacity; and by which it is capable of taking cognizance of the outer world; but even under such conditions it can excogitate, and create new and unknown states, and therefore it furnishes the strongest evidence that it has an independent existence.

After these preliminary remarks, we will submit some curious dreams, to show that particular kinds of food may have a special tendency to suggest the rude material of the nocturnal vision.

The first time we entertained this view of the subject was in consequence of the following incident:—

A gentleman who was not in the habit of eating supper was induced to partake of some richly-stewed halibut, as that fish was a favourite kind with him. On his return to his house he read for two hours a very interesting essay, so that it was past one o'clock before he retired to rest. "To rest the body (as he said), but not the mind." For, although he fell into a sort of slumber, his busy thoughts were occupied by the subject of man's abstract rights and political wrongs. After this almost waking-dream, for it referred to his previous reading, his tired faculties became more intensely clouded, and soon the busy world, with all its follies and its crimes, its wisdom and its errors, became oblivious to him. After a time he had a dream, most painful yet most curious. He was walking with a niece (a young girl about twelve years old) by the seaside, on a most picturesque coast, and finding himself greatly fatigued, he determined to rest himself on a ledge of the granitic cliff. The place he chose, from its abrupt slanting sides, was like the roof of an ancient house. He remembered that he selected the slope nearest the land-side, but from some cause which did not appear in the statement, he ventured to crawl over the crest of the ledge, and to his horror found himself sliding downwards to the rough and boisterous sea, which was at the time lashing the base of the cliff in a most savage manner. His sensations were indeed agonizing. Cold and clammy perspiration enveloped his whole body, as if he had been clothed in wet garments, yet he made an effort to save himself. He called to the little girl to climb to the summit of the ledge immediately, and let him take her hand. She did so with great promptitude, as if conscious of his imminent danger. He could not recollect how long he seemed to hang in his perilous situation, suspended between life and death by a frail thread; but he distinctly remembered that he reasoned on the improbability that he could hold on much longer, and that as he grasped his little relative spasmodically, he dreaded that he might also drag her to instantaneous destruction. However, nerved by his terrible predicament, he determined just at the crisis when his painful conjectures would probably be realized, that he would make one desperate effort to save the child and himself;—he did so and succeeded, yet when he got on the safe side of the ridge he fainted. The latter sensation was merely the transition from the dreamy to the waking state; if the painful condition he was in could be called so, as he continued under the influence of his ideal terror. His body was very feverish, and his heart was so much disturbed as to induce a sensation of sickness.

The heated state of the skin was evidently the result of the rich fish he had eaten (as fish by night has more or less a feverish tendency), but that the food should have been suggestive of the scenery, as indicated by the incidents of the dream, leads to a very curious subject of inquiry for the psychological student.

Sir Charles Bell said, on one occasion, "that there are no accidents in nature." This truth may be applied to the investigation of nocturnal visions as well as to physical phenomena. For whether the active soul experiences pleasurable or painful emotions, these emotions depend partly on real causes, and cannot be considered as either adventitious or arbitrary. This opinion we hope to confirm in the evidence about to be submitted. Two preliminary considerations present themselves in our investigation, namely, that not only shall we find that the kind of food may determine the events of a dream, but often also that the pursuits of the dreamer will tend to give his dreams some speciality.

For example, we knew a medical man who after attending a lecture was very hungry, and, contrary to his usual practice, ate a hearty supper of rich fish with a stuffing composed of liver mixed with savoury herbs and spices, and some stimulating sauce. When he went to bed he had a rather dry and feverish skin, but he soon fell asleep and dreamt the following curious adventure:—

He said, "that he thought he was travelling in a very hot country, which abounded with a number of poisonous serpents, particularly the 'cobra capella,' and that having the opportunity, he determined to dissect some of them, and that he seemingly did so with impunity, until he proposed demonstrating the structure of the secretory glands which generated the poison. These he traced to the lower surface of the tongue, and when in the act of carefully pressing these glands to ascertain the colour of the deadly fluid, a little of it spirted into his face and caused him great pain. The sense of his dangerous position awoke him, and he was highly gratified to find merely a little eruption on his nose and face, evidently induced by the rich food of which he had partaken the previous night." And he added, that indigestion was invariably relieved, in his own case, by eruptions on the skin.

We select another instance to prove that particular food not only gives the speciality of the dream, but also in this case revived impressions of the past, and gave them all the vividness of reality.

"The other night I was very hungry and was induced to eat supper, and added to the outrage of partaking of cold roast beef, the folly of eating about a dozen pickled onions! But as I felt very comfortable and had a long way to walk, there was not any misgiving that for so doing any great punishment would result.

"By the time I reached home it was at least two if not three hours after taking the unwholesome meal, and soon afterwards I went to bed. I felt somewhat uneasy, but it was not very long before I fell asleep. Soon afterwards I dreamt that for some offence of etiquette I was doomed to eat '*sour-crout*.' Now it so happened that this sort of pickled cabbage always caused me most painful nausea even when smelling it, so that I had never been prevailed on to taste it. Nevertheless, in the vision, it seemed that great pains and penalties awaited me unless I abided by the unchangeable fiat of the judges.

"I fancied myself brought into a large room, in the centre of which was the reeking *crout*, and what with its acid fumes and its fatty smell,* I was almost overpowered, and I experienced a sense of gastric resistance. Still I made the effort to eat, but every mouthful which I endeavoured to swallow was immediately rejected, and the painful and sickening sensation increased at every attempt to retain the abominable stuff. My situation was indeed pitiable; and the perspiration trickled down me from the agony I experienced. It was a battle between *sour-crout* and the stomach, and the struggle was desperate, but the stomach conquered, and I awoke.

"My mouth was filled with acidity and the disagreeable taste of the onions, which conjointly rendered my sensations most unpleasant. This condition of the mouth had evidently suggested the dream, and an early antipathy for fermented sour cabbage was reproduced with as much vividness as if the *crout* had been positively served up, and coercion had been used to enforce its being eaten."

This individual was advanced in life, and had not seen or smelt the sour-crout since his boyhood, yet its odour appeared as disagreeable to him in his dream, as it would have been in his wakeful period.

We will now relate another amusing dream, suggested by the nerves of taste, which occurred to a lady, a friend of the writer, and which furnishes some speculative thoughts to the psychologist.

It is not often that we specify the age in these cases, but it may be incidentally noticed that the dreamer was about fifty-seven, with a nervo-bilious temperament, and of the most simple habits, never indulging in any kind of alcoholic beverage, although she was not pledged to abstain from them.

Mrs. —— had a slight cough, and one of her interesting daughters brought her some barley-sugar to suck when in the act of lying down, or in case the cough disturbed her during the night. She had, on the occasion we are about to relate, taken a piece of this *sweet* "medicine" into her mouth, and soon afterwards fell asleep, with the confection undissolved.

She dreamt that she was a very little girl, and that she was spending the evening in a juvenile party, at which, besides tarts and jellies, there was added an abundance of sweetmeats of the most delicious kinds. She felt superlatively happy, and what contributed to ensure this satisfactory state, all her early associates were present. These friends of her childhood appeared, the same laughing girls and boys, and were apparently undisturbed by care or annoyance of any kind. Yet she seemed to have some under-current of misgiving, for many of these *spectra* had been dead for years, and others she had not seen since

* In Germany sour-crout is usually stewed with fat beef; hence his dreamy reminiscences were the impressions of his childhood.

their school-days, when she herself was but a little girl. But these reflections did not affect her, for she fancied herself entering into all kinds of childish sports and pastimes with all the glee imaginable, and so she continued to laugh with those around her until she awoke with a smile on her features; and so brief was the whole period of the dream that her daughter (who had been talking to her) had not left the bed-side, for she had not been asleep more than a few minutes.

When Mrs. —— related this vision to us, she said, "With my restoration to perfect consciousness I could but wonder how any such illusion would so engross the mind, and appear so very, very vivid and real." But the explanation as to the predisposing cause was simple, for when she awoke she was still sucking the barley-sugar. And there is every probability that as she was going to sleep the passing idea occurred, "I am like a little child to go to bed with sweetmeats." This suggested to her mind the train of pleasing thoughts. For when the world was closed on her unconscious senses, her soul thus untrammelled revived the scenes of her juvenile days, and called forth from the shadowy past her former associates, companions who had been forgotten during her more matured age with its hopes and fears. Anyhow we regard this dream as revealing one of those beautiful facts indicative of the soul's independent functions—that there are times when there is an obliviousness of all mundane trials, and the mind is imbued with sentiments of purity and innocence, and yet it experiences the most exquisite emotions, and however brief the time, it is enabled to feel perfect happiness!

We have had related to us many other similar kinds of dreams which corroborate this passing speculation. But instead of reporting them, we will submit another species of dream from its psychological interest. We select one instance, in order to show that the mind during sleep seems under some circumstances to confirm a conjecture which during the hours of wakefulness may be only surmised as a mere transient reflection. A friend of ours, a man with an active temperament and a sensitive brain, was devoted to modelling and sculpture as a pursuit, and to literature and science as relaxations, and to his other qualifications he was said to be a most skilful physiognomist. But he rarely ventured to act on his knowledge of character from mere facial indications, lest he might inadvertently commit an injustice. To use his own words, "though he had faith in the science, yet he was conscious how much man was the creature of education, so that probably the most degraded might, by a train of propitious circumstances, not be what he seemed to be," therefore, however meanly he thought of any one, he never refused to do him a service.

These particulars are essential to appreciate a dream he had, which we shall relate in his own words.

"It was the latter end of June, 18—, when the atmosphere seemed saturated with electricity, and day after day there was heavy rain with thunder and lightning, and yet so great was my sense of fatigue that I determined to take a stroll to refresh myself, having been mentally hard at work for many hours. During my walk I called on some of my wealthy relatives, and was urged by them to stop to take some tea, which I declined, and returned home about nine o'clock. After enjoying my meerschaum until eleven, I was induced to partake of a slight fish supper. It was some time after 'the witching hour' that I went to my bed-room, but not to sleep, for with my pipe I sat watching the beautiful coruscations of electric fluid for an hour or two, until 'tired nature' inclined me to rest, and I was soon in a sound sleep.

"Early in the morning when I awoke, my mind was impressed with a most vivid dream, so vivid, indeed, were all the incidents, that it must have occurred just before awakening from a rather more than usual long slumber.

"My dream is indeed a curious lesson. Its philosophy I'll leave for the reflection of some profound student of mental science. Methought that I called at the house of Mr. Sneak, and that he showed me an order for some bronze figures, and I suggested to him a few ideas on the way in which they should be grouped, when he shook me by the hand, saying, 'Thank you, my dear sir; you are indeed a capital fellow, your hints are admirable,' &c.*

"I proposed a walk, and Sneak consented to go with me, and as the night seemed gloomy he took the precaution to take a mackintosh and a small umbrella, with neither of which had I provided myself, and as it soon began to rain I proposed to go into a tavern for shelter. He refused at first, and I said, 'Then I'll bid you good night, otherwise I shall be drenched to the skin;' so then Sneak accompanied me. We entered the part of the hotel dedicated to the family. And as they were at tea, we were asked to join them. Everything looked so clean and bright, even to the copper tea-kettle boiling at the side of the fire, that we accepted the invitation. Sneak, to show his affability, went unsolicited to bring the boiling kettle to the lady who acted as the presiding genius, but he soon dropped it, as the handle was very hot. Though he had caused much discomfort by the accident, yet with great politeness he was asked if he had hurt himself: he did not answer, but looked at his hand, coloured up, and then became very moody when he took a seat. I sympathized with him and proposed a dredging-box to flour it, which he declined in a rather pettish manner. He, however, ate some buttered toast and drank some tea, and then again looked very sorrowful at his hand and cried!

"I offered to procure him, when the storm ceased, some cotton-wool, but he actually refused my services, saying he would go and get some and return very shortly; but an hour had passed and Sneak was still absent.

* The narrator told us, in parenthesis, that Mr. Sneak had often betrayed his insincerity; and that he was literally "all things to all men," and had, besides, the bad habit of "throwing the hatchet,"—*anglice*, "lying."

"I paid the score and went to see an eminent artist on whom I had promised to call. The servant said he was engaged. 'Tell him a gentleman wishes to say a few words to him, and that he will not detain him but a few minutes.' She did so, and he came, flushed and somewhat confused, to speak to me. Mistaking his manner for chagrin that my visit had been so late, the cause for which I briefly explained, when he said in his frank manner, 'Why, my companion is Mr. Sneak, and he has just been abusing you, declaring that he'll cut you in future; that you are a nincompoop, and that through you his accident occurred.' 'Why, surely you are joking with me; Sneak cannot have dared to have misrepresented things in such a manner.' 'But he's the very double-faced fellow to do this!' 'Well,' said I, 'you like a touch of nature, and if you please we will unmask him; I will go away, and soon return, so bid your servant show me into your room unannounced. Then you will see how he will fawn and cringe.' As arranged, I walked into the room *sans cérémonie*, and Sneak coloured up, then turned deadly pale. His hand was in a sling, formed of a silk handkerchief. He then apologized for his rudeness in leaving me; but he continued, 'You are such a noble fellow and so forgiving that I am certain of finding a lenient judge,' &c. My rough artistic friend turned to him in a rather sharp manner, and tauntingly asked, 'Do you, Master Sneak, say these fine things to the nincompoop?—to the bore you intend to cut?'

"Poor Sneak was chopfallen, and particularly so when Mr. —— said, pointing to the door, 'You had better go, Mr. Sneak, and not wait to be kicked out;' and was in the act of suiting 'the action to the words,' when this vain fellow rushed from the room, bellowing like a town-bull, and this last incident awoke me.

"What a 'yarn' the brain had spun, simply from refusing the tea, the weather's inclemency, and from having heard that Sneak was rejected from the 'People's College.'"

But there is a moral even in a dream, for our informant added, "That Sneak is just such a sort of personage, that he invariably spoke against his acquaintances behind their backs, and was affable even to servility when they were present."

We remark further, that this dream has certain psychological aspects. 1. That it is evident that the dreamer had previously suspected the sincerity of Mr. Sneak. 2. That in his sleep a scene is produced calculated to expose this defectiveness of his character. 3. That the brain not only exhibited its dual function by the presence of the "artist" and "Sneak" at the same time, but the *egoism* of the dreamer (which also takes part in the mental drama) gives the cue to the other *dramatis personæ*, and thus positively brings out in fine relief the respective peculiarities of each.

It is not essential that at all times we should partake of a particular kind of food to induce the dreamy process; this inducement may also result from a disagreeable or pleasant taste,* which, being perceived by the partially awakened consciousness, may tend to excite certain trains of thought which are either naturally associated with, or which are suggestive of others, the proximate cause being positively disconnected. We select the following example because it possesses certain psychological peculiarities :—

"A medical gentleman, who was in the 'sere and yellow leaf' of life's seasons, had partaken of supper, in which eggs in some part formed a portion of the repast. He also took a glass of bottled stout, an excess he was guilty of not more than thrice a year. Yet he says that he felt well when he retired to bed, although his skin was somewhat feverish and his mouth rather dry. The latter he attributed to having smoked a cigar. He relates that he was not long before he fell into a profound sleep, but some time before he awoke he dreamed that he was an usher in a school, the proprietor of which almost starved his scholars, and put him and the other functionaries on 'short commons.' This dream-created 'Squeers' gave them all what he called 'a late tea,' but not any supper, and he said he did so because the last mentioned meal was like taking with prepense a deadly poison into the system. Sometimes, however, he relaxed in favour of his assistants, from a conviction that they were acquainted with his own excessive indulgence in 'creature comforts' in the form of supper, and he professed that this meal was *qualified* in its poisonous tendency by a few glasses of stiff grog, and he whispered his 'better half' that he must make an occasional exception to prevent a mutiny.

"The dreamer thought on this occasion the proprietor had invited the classical tutor and himself to partake 'the unwholesome meal,' which consisted of boiled eggs, but which eggs were scarcely warmed in the water; and as the night was cold and the room cheerless, he (the dreamer) made up his mind to preserve his own eggs and have them better done, even though it would be so much later before he could eat them. He therefore managed to conceal them in his hat, placing over them his pocket-handkerchief, and being pleased with his success, he finished his comfortless meal.

"It seemed, however, that the 'master' had observed the trick and determined to thwart it, so he asked the dreamer to go to his study for a book, and during his absence crushed the half-cooked eggs, without disturbing the temporary covering. Our dreamer returned and took his hat, and as he went into the street put it on, when to his horror and annoyance the glairy fluid streamed over his hair, eyebrows, eyelids, and almost blinded him. This would have been to him sufficient annoyance, but he fell in a rage when he heard the coarse, vulgar voices of the 'master' and his selfish brood laughing at his mishap, and insultingly shouting 'serve the humbug right.' 'Take care of your eggs,' 'take care of *the* eggs;' and then another and

* As in the case of the "sour-crout" and "barley-sugar."

another peal of laughter, until his indignation was roused to frenzy, and in the act of denouncing their worthless natures he awoke."

To our query he replied, "that he recollected distinctly that he had a most unpleasant taste in his mouth, like rotten eggs (probably induced by the beer and cigar, which had rendered him bilious), and that for the whole adventure of his vision he could only account by recollecting a similar incident which had occurred to an acquaintance of his when they were boys together, but that he had entirely forgotten the circumstance until its reminiscence was renewed in his dream, but that the talismanic influence of Memory had omitted the actual hero, and fixed the whole adventure on himself. This latter consequence he attributed correctly to the state of his mouth.

This proves that, however absurd or irrational, or actually untrue a dream may be, that it is still governed by what has been termed "the association of ideas." For in this instance we may mention that that part of his sleeping adventure, "when he was in a cold, cheerless room," was occasioned by his feet being out of bed, the window being accidentally left open, admitting a strong current of wind, which lowered the temperature of his whole body, so that even in his sleep he must have actually shivered.

Sometimes we may trace the connecting links from which the events of a dream may have been fabricated; and on other occasions we may find a strong admixture of truth and romance, so blended as to leave the mind unsatisfied with any attempted solution to the mental riddle. The following dream, related to us by Mr. Y——, will illustrate our meaning. He says that he attributed his dream to having taken a cup of tea, a beverage he had abstained from for some years, as it invariably produced a painful action of the heart, and sensibly accelerated the circulation of the blood; we will give his own words:—

"On —— I paid a visit to a literary friend, accompanied by two of my daughters, and as usual we spent a most delightful evening. Our host was a man of versatile genius, being a linguist, artist, mechanist, and highly musical. When the members of the family took tea my daughters joined them, but they were greatly annoyed at my declining to do so, and after repeated urging I consented to take one cup of that 'which exhilarates without intoxicating,' and its visible effect almost immediately after was manifested in an extraordinary flow of animal spirits, and a copiousness of expression almost like inspiration.

"In the course of the evening our host played the piano-forte, and he was followed by one of my children, who was praised for her execution and brilliant touch. And whilst this concert was going on, I walked into another room to converse with the amiable and intelligent lady, and whilst doing so we heard the sonorous and rich voice of her husband chanting sacred music. These floating sounds suspended our conversation, and when we resumed it, I distinctly remember saying 'that our friend's varied talent and fine intelligent face would have made him a dangerous priest to confess susceptible girls,' at which my companion said, laughingly, 'So I think!'

"We returned home about twelve, and went to bed, but the tea prevented my sleeping, and besides, my mind was more than ordinarily vigilant from the different sources of excitement to which I had been exposed. After tossing about an hour or two in no very pleasant mood, I fell into an uncomfortable slumber, and dreamt that a fine, portly, good-looking priest visited us, and that from his manner to my wife he aroused my anger and jealousy. I denounced him as a sensualist, unworthy of his sacred profession, and mentally proposed, as an experiment to test the accuracy or error of my suspicion, that I would intimate my intention of being absent from home for some days; but at midnight I returned, and entered my house by a private door, the key of which was in my sole possession. Noiselessly I proceeded to my bed-room; it was locked; and as I turned in a moody spirit to consider the best way to act under the circumstances, I fancied I saw the figure of the priest glide towards the bed-room of one of my daughters. I followed, and beheld the villain in the act of moving the bed-clothes from the sleeping innocent being, and struck him with my bent fist, so that his whole person was covered with blood. He looked like a priest officiating at the *taurobolium*,* and regarding me with the expression of a demon, he raised a glittering dagger to plunge into me. It was the work of an instant. I sprang on one side, and then suddenly returned and gave him a desperate blow on the temple, and he fell flat on the floor, and the noise awoke my whole family, some of whom, and amongst them my wife, rushed from their beds to ascertain the cause.

"A few words of explanation induced my good partner to tell me that the wretched priest had attempted to seduce her, but on her threatening to expose him, he promised to leave the house, but she added, 'he had evidently concealed himself, and has met the punishment he merits.' We gave the alarm by springing a rattle and calling loudly for the police. Soon five or six made their appearance, and we gave the sanctified wretch into custody. As the officers were securing him he recovered his consciousness, and begged he might be spared the public disgrace, and urged that I should drive the dagger (which he still held spasmodically in his hand) into his heart rather than suffer his conduct to be exposed. In order to arouse in me a spirit of revenge, he confessed 'that when he found my wife invulnerable to

* "The TAUROBOLIUM of the ancients was a ceremony in which the High Priest of Cybele was consecrated, and might be called 'a baptism of blood.' In this dreadful and sanguinary rite, the high priest to be inaugurated was introduced into a dark excavated apartment, adorned in his vestments. Above this apartment was a floor perforated in a thousand places like a sieve, through which the blood of a sacred bull, slaughtered for the purpose, descended in copious streams on the enclosed priest, and in which bloody shower he bathed his hands, cheeks," &c.—Abridged from the Poet Prudentius, and cited at length by Banier "On the Ancient Sacrifices."

his sensual proposals, he determined to pollute my favourite child.' This did rouse my ire, and as I awoke I was screaming, '*Take him away!*'

"My heart was beating in a most painful manner, and big drops of perspiration bathed my forehead. And yet in all probability the whole dream did not occupy the time that it has taken to relate it."

We may remark that all this mental suffering was attributed to the tea, which had affected the nervous system, and fearfully increased the action of the heart, which he said felt painful for some days.

We believe that much information would be derived regarding physiological and psychological science, if men would note down any peculiar states and sensations induced by various disturbing influences; and hence we record the following as worthy of some reflection:—

"A gentleman was requested to write an article on 'gas' for a periodical, in which he was to treat of the subject. He therefore was occupied for some days with studying the chemical composition of gas, the best mode of purifying it, and the advantages and disadvantages of its use as considered with respect to bodily health.

"Having satisfied himself with his different experiments, he had decided on writing his article the following day, but prior to going to bed he had eaten some raw apples and bread, and drank merely cold water. He says, 'Although this was an abstemious meal, yet before retiring to rest I was somewhat annoyed with flatulency, which, although rendering me very uneasy, yet from being very tired this did not prevent me falling into an intense sleep. But I had a dream which so thoroughly roused me, that the 'sweet restorer' seemed banished from mine eyelids.

"'It seemed to my busy thoughts that I had invited a number of scientific men, and introduced to them not only the various opinions entertained on the use of gas, but also submitted to these *savants* a series of experiments that I had tried, for what purpose my *egoism* did not note. The whole assembly regarded me with astonishment, for I caused to issue from my mouth, spontaneously, streams of colourless gas, which on being ignited, gave out not only a clear and brilliant light, without the slightest odour, but was so volatile that it spread out widely with a fan-shaped flame, which seemed to increase and render the atmosphere most lucid.

"'One of the learned party exclaimed, 'This marvellous display is an instance of 'spontaneous combustion;' let us observe calmly the results, and endeavour to ascertain if the individual is entirely consumed; for in studying this phenomenon, what signifies sacrificing the life of an individual?' It is a curious fact that I did not seem alarmed, but only experienced some anxiety to know the nature and quality of the gas. I tried repeatedly to speak, but at each effort greater volumes of the luminous agent poured forth with greater rapidity, and the light gradually grew stronger and stronger, and too vivid to look at.

"It amused me to see the learned jury remove from time to time further away, merely remarking it would have been better if, instead of a man's mouth, it had issued from a half-burner.

"Every now and then my own efforts increased to ascertain something more definite by the smell. At length I jerked out hy——, hy——, hy——; but the word seemed to stick in my throat, although I felt assured it was hydrogen gas, and used painful and grotesque expressions in the endeavour to communicate the fact, and the whole party burst into a roar of laughter at my awkwardness. This ignorance on their part forced me to attempt a more concentrated effort to explain, and in this effort I awoke.

"And soon there seemed a solution, by the fact that I was oppressed with flatulency; and the impotent attempt I had made to expel the flatus had occasioned the latter portion of the dream, and the origin of the whole train of thought was evidently traceable to my mind being occupied for some days previously with the subject of gas, which was so curiously mixed with the whole adventure."

We would now remark, that there is presumptive evidence to indicate that, whatever may be considered the predisposing causes of different dreams, we may often discover, during these nocturnal vagaries, some predominant faculty at work in the mind of the dreamer, which faculty seems to give its reflex influence not only to the mind during sleep, but also to the thoughts, even in a state of perfect consciousness.

In the instance to be cited we shall find that the speciality of the dreams was induced by the particular food the dreamer had eaten, although the amusing sleeping adventure indicated a strong tendency to the grotesque and the ridiculous.

We were at an evening party at the house of one of our relations, when we were accosted by a comic-looking gentleman, a medical practitioner, whose expression was so naturally humorous, that he would have excited a smile even in a suffering patient. He said, "I am told you are an interpreter of dreams, and are acquainted with all the *arcana* of these curious phenomena. Will you explain one I had recently? We expressed a wish to hear it, and he continued thus:—

"Methought I was announced to lecture on some new kind of 'revalenta' food, which was cheaper than bread, and combined the rich flavour of meat and poultry. A vast mob had collected to hear me in the market-place—a most fitting spot to discuss the merit of different kinds of food. I had not made my toilet—but this did not annoy me, as my auditory were all *en deshabille;* but I had omitted to bring the MS. of my discourse. So I made my apology, promised to be back within an half-hour, and then they should have my revelation to repletion. Three cheers encouraged me, and full of spirits I started on my errand.

"But somehow or other I could not again find the market-place, but continued wandering about until it was nearly dark. Everybody whom I asked merely stared or made faces at me; and when requested to tell me 'the way I *should* go,' replied with a coarse laugh, with the vulgar saying, 'Does your mother know you're out?'

"At length a Turk, in his full costume, proffered his services, and led me to a rudely-constructed bridge, composed of long cylindrical bones, imbedded in some animal matter of a soft and unctuous kind. My guide suddenly withdrew his arm, and in pantomime bid me proceed. This was no easy matter; but I should have toiled on, if I had not suddenly been assailed by invisible fiends mockingly crying, 'Fish! fish!' 'How do you like fish, Doctor?' 'Stick to the fish; that's right, Doctor!'

"These are but a sample of the taunts with which they pelted me, and made me feel worse than if I had been put in a pillory. My annoyance was greatly aggravated from being literally glued to my fishy path. Yet in the intense darkness it was impossible to say what was the actual substance. Fixed like a prisoner in the stocks, I had no other alternative than to bear the annoyance until the daylight should reveal my own position.

"When the sun burst forth from his eastern couch, my astonishment was indeed great, as the whole cementing medium which had so effectually attached me to itself proved to be decomposing salmon, arranged in layers over a vast many kinds of animal bones. Angry with my not *very* agreeable situation, I felt still more so to think such a beautiful fish should be used for such a purpose, and then I made a desperate effort to get away, and immediately awoke."

This dream was indeed both curious and amusing, so we commenced a rigid "cross-examination," and ascertained two probable suggestive sources for the kind of fish, and the outraged experience that it should be so wasted, contrary to all taste and gastronomic propriety.

The doctor had been reading a paragraph in the *Medical Times and Gazette* (June 20th, 1857) giving an account of a *conversazione* held at St. Bartholomew's Hospital, where, among other objects of interest, there were shown the *ova* of salmon preserved in gelatine, and some account of the artificial reproduction of this rich and royal fish from similar preserved specimens was given.

We also had the information that he had been unable to take his dinner, and he had therefore taken tea at the house of a friend and patient, a foreign gentleman, who ordered up some fine slices of fried salmon, and after satisfying his visitor on this solid fare, there came some rich smoked salmon; the latter, he said, was so very rich and fat, that it seemed to melt in the mouth, so that he said these rare luxuries tempted him to eat a heartier meal than was judicious.

"At all events," we said to the dreamer, "there is a clue to explain the notions of your 'lecturing on food,' and of the salmon pathway. These were floating ideas which passively exercised an influence on your general thoughts. Nay, it is not improbable that, whilst eating the rich smoked salmon, with your witty tendency, that you might have had the whimsical notion 'that a material so very soft would be nice to walk on,' " &c.

When we had given this solution, the doctor said, quizzically, "Your explanation is very good, but whence arose the suggestive clue that a Turk should have offered to guide me through my difficulties, the rogue and hypocrite leaving me embedded in no very agreeable position."

The turbaned Turk was simply a stray thought, a sort of mental parenthesis, and originated in your annoyance at yourself for tickling your palate even to repletion; and when the first symptom of annoyance was experienced, it is not at all unlikely that you should say, "I am worse than a Turk," because he could not, with orthodox authority, "take a little wine for his stomach's sake," if you could. "But I did not," said the doctor; "I took a little brandy instead;" and he laughed in a very merry manner, and then said, with a more serious expression, "There are indeed moral lessons in dreams!"

We should not be disposed to give a mere collection of curious dreams, if it were not that they aid in furnishing data to explain, still more than has yet been done, the philosophy of many mental phenomena. Amidst many of the extraordinary anomalous and inconsistent adventures during the state of partial consciousness, there is something like a law which regulates them; and when there exists any special exciting cause, as food for example, if the dream does not actually develop its incidents in connexion with the viand which had determined the imperfect rest of the brain, yet it will often be found that the soul seems conscious of the source of the disturbing agent, and reproduces incidents and facts which in the perfect waking state had been forgotten.

We submit the following instance as an illustration of the view just intimated. A scientific friend writes thus:—

"After lecturing the other night at the Philosophical Society of ——, I took, what to me is an unusual beverage, a cup of strong coffee. It rendered me excited and indisposed for sleep. This was the more annoying as I was suffering much from chronic constipation. There was no remedy but patience, and I had to brood, or regret that my injudiciousness had brought the severe punishment of extreme wakefulness, with all its irritating consequences. However, after great restlessness for hours, sleep overpowered me; but in that sleep, alas! my mind was more energetic and morbidly active than in my waking state; and in its discursive vagaries I passed through many countries, consulted numerous physicians, climbed mountains, or tossed about on rough tumultuous oceans, evidently restless in this pseudo-sleep to discover where I might find some mitigation of my feverish condition.

"After a variety of adventures and hair-breadth escapes, I arrived at the fashionable B——, for the purpose of consulting Mr. M——, a very old and skilful practitioner in that town. It may be remarked, incidentally, that he was in the habit of giving his patients some moral advice with his physic. In my case, however, instead of his usual courteous and affable manner, he abused me most soundly, and concluded with saying, 'You ought to suffer, if you are such a slave to grovelling appetites as to have no control over your actions.' After expressing my sorrow for this supposed delinquency, he answered in a most pettish manner, 'Fools are always doing that which brings them sorrow; and a learned fool is no exception.' This rude remark fired my temper, and I replied, 'How can I be assured that your opinion is so very profound?' when he aimed a blow at me with a pestle he had in his hand. And then I taunted him with his own wise saw, 'Fools are always doing that which brings them sorrow, and the disciple of Esculapius is no exception!' Scarcely had these words escaped me, than his son, wife, and footboy all assailed me; one with a scalpel, the other with a dissecting-saw, and the latter with a huge physic-bottle, and in the midst of the *mêlée* I awoke.

"Reflecting on what could have induced this amusing dream, I remembered that Mr. M—— once had recommended me to abstain from coffee, as he said that it was very bad for those who had constipated bowels. This opinion had been given some years past; so that with an imperfect consciousness that the coffee had been the disturbing cause, arose the reminiscence and the train of incidents it induced.

"It is not the least curious circumstance to mention that, although I had not seen Mr. M—— for some years, yet his individuality and quaintness were preserved most vividly in the dream."

We may mention in this place that, although a dream may be the result of particular food, still this may only give the direction to the adventures in the nocturnal vision, and yet the events themselves may be still traceable to the peculiar condition of the body from the nature of the food eaten.

An old gentleman related to us the following dream soon after it occurred:—

"One night we all supped at the house of a rich and worthy foreigner, who gave us most delicious fish, meat, pastry, and fruits. For myself I luxuriated on some fried salmon, though not to excess, and returned, home about eleven o'clock. The night was very beautiful, but never did I feel more comfortable. But when at my own residence, I experienced some slight feverish symptoms. My hands and body were hot and uncomfortable, and yet I had not drunk either wine or spirits. I retired to bed, and my feverish state increased greatly, for my active brain busied itself with all kinds of speculations.

"Tired and jaded, at last I fell into a heavy slumber, and was busy at the sea-side with some of my children. We were walking on the beach, which was covered with dank sea-weed of many colours, and some rare and new species of flexible coral. We found it difficult to move, and with every effort to retrace our steps, we met with most annoying and uncomfortable resistance. In the midst of all these annoyances the clouds became black as ebon, and the wind whistled a dirgic melody, the sea ran mountains high, and a storm of a terrific character seemed gathering, and the spray and surge dashed over us with the unnatural clatter of so many brawling creditors. Our juveniles turned pale and cried, and yet, amidst all this 'war of elements' I seemed inclined to examine some of the specimens scattered around us. 'Pray let us go,' was said and repeated; still I heeded it not, but with a pocket lens was examining what seemed, from its arborescent form, a flexible coral, when to my surprise it proved to be *scales* of the salmon agglutinated together in a most graceful manner. The lightning became then most awful, running on the beach and the sea, which gave the latter the appearance whilst the glare lasted of a fiery ocean. Then I also desired to leave, and soon without any apparent volition we were safe in our apartments. After a time there was a slight lull, and out I sallied in my wet garments to collect the salmon-scale specimens, having lost those I first collected; but scarcely had I stopped to take some of them, when a huge wave rolled over me, filling my mouth and ears, and so stunned me that I awoke. My condition was anything but agreeable — skin burning; my mouth, instead of being filled with water, was dry and parched; and my temples throbbed in a painful and unpleasant manner."

This dream was induced by the "dainty dish" on which our friend had supped. And even in his sleep he had a presentiment that such was the case. Hence the notion of the seaside, the pleasure of examining the bright salmon-scales, and the state of the buccal cavity. That he should have supposed his enthusiasm would have rendered him calm in such danger, may be in some measure the fact, as his taste for natural history is a passion; but he certainly would never have permitted any of his own inclinations to expose those he loved to suffer from terror, or even any misgiving of safety, without making every personal effort to disabuse their minds. His whole mental state in the dream was similar to that manifested under the influence of fever. He jumbled together many incidents occurring at different times, and blended them so that in their connexion they furnished a rather curious adventure.

We select a few examples of the dreams which have occurred during after-dinner naps, for the sake of the curious composition and rapid progress of the events observed in them. Some of these dreams are of recent occurrence, and others are of older date.

The narrator of the first dream we propose to give had, a short time previous to its occurrence, been writing an article "On the

Treatment of Ticket-of-leave Men," in which he deprecated the folly of giving them their liberty without ensuring them employment; and he expressed his great indignation that the police tracked their paths, and thus prevented them from living by honest means, and in a measure forced them back again to a criminal career! This gentleman had eaten a hearty dinner and taken up a newspaper, when he read of some trick in reference to the sale of certain jewels, and with a very confused notion of the affair he fell into a profound slumber, in which the following dream occurred. He thought that he had called on some friends who congratulated him on his humane efforts, and they drank his "health," calling him "the servant of Mercy." And although he felt gratified, yet soon there appeared much hurry and bustle, as when coach passengers hurry from the table to regain their seats, and all seemed to have evanished. And then another incident excited his busy brain, and we will report it in his own words, literally, because of its peculiarity :—

"I thought I was living in the neighbourhood of a foreign court, situated in a very beautiful locality, and that in the town there resided a hard-working, clever jeweller, who was considered to be in difficult circumstances. But to the surprise of everybody he suddenly made a better appearance, and in consequence obtained some employment from the royal family. Yet people marvelled at this man's success, and speculated on its probable source. It happened (but how I have not any recollection) that I ascertained that he had found a very valuable diamond ornament, which his poverty tempted him to break up and sell the stones at some distant place, and in order to avoid detection he melted the gold with some he had by him. Whilst he was attending to the latter process his agitation was so great that he upset the crucible, so that its contents fell into the furnace, but he collected as much of it as possible, and afterwards discovered that he had lost four pennyweights of the precious metal. In the midst of this annoying circumstance two officers of justice arrested him, and he was taken before the king. His Majesty was a very jolly but imperious personage, and acted as the supreme judge. The prisoner was charged with the fraud, and the accuser said there was every proof of his guilt, for he had sold the diamonds. The poor jeweller looked at first rather more astonished than frightened, but soon he recovered a dogged manner and refused to answer any question. His royal judge lost all patience, and desired he might be put in 'the pillory.'

"This threat seemed to arouse the delinquent, for he answered in a somewhat angry and insolent tone, '*Pay me first for my four pennyweights of gold which I lost in melting the ornament!*'

"'What does the idiot mean?' said the judge. 'Pay me for the gold,' answered the disgraced jeweller.

"The king became excited, not with rage but laughter. 'The fellow's a fool,' shouted the judge. 'Ha! ha! ha! Don't you perceive, you dolt-head, that you confirm your guilt?' 'Will you (answered the enraged criminal in a threatening manner) pay me for the gold? For the melting of your ornament?'

"'Take the idiot away,' shouted the king, 'for he must be *mad* as well as foolish. Put him in a madhouse for life!'

"The whole scene amused me to think that a man who seemed so simple as this poor jeweller did, should have hit on such a clever finesse to get out of his scrape, and become well cared-for for the rest of his days.

"And his family were also saved, because of the sympathy which the court felt for the supposed malady of the delinquent. For myself it had a similar effect as a comic scene in some racy comedy, for I was tickled at the way he confounded the judge, and awoke laughing most heartily.

"As a curious instance of the activity of the soul, when the greater number of the bodily and mental faculties are oblivious of all sensible impressions, I may mention that the whole of this incident (which is much abridged in the narrative) had actually taken place in less than three minutes, for just before dozing I looked at a clock on the mantelpiece, and the whole was concocted without any apparent data for the incidents."

We have mentioned that this gentleman had written a paper "On Ticket-of-leave Men," but he had not that day been either speaking or thinking of these men; he remembered having enjoyed his dinner very much, and after he had said grace, he observed to some of his family, "I don't envy a king!" From this simple phrase, and from the fact that his mind had been preoccupied with the problem how to improve criminals, we obtain a clue to the direction which his mind had taken in the course of his dream.

Another friend mentioned to us that from a simple remark made after dinner, prior to his falling asleep, a dream was induced which was in some degree a reminiscence of his boyish days:—

He said that he had eaten heartily of some roast mutton, with other things, for his dinner, and as this was his favourite animal-food, and being hungry, he had eaten with great gusto. One of his daughters declined any meat, as she had an antipathy to mutton, and our dreamer remarked, 'I am sorry you are not, like myself—a carnivorous animal!'

After the dessert he sat in an easy chair, with his feet on an ottoman, and fell into a sound sleep, in which he dreamt that he was partaking of a most delicious dinner all by himself, except that there was a large, shaggy, savage-looking dog under the table, who kept constantly rubbing himself against his legs, and in order to avoid this annoyance, he threw the animal many a precious morsel. But, instead of satisfying the canine intruder, this civility only made it more importunate. When, therefore, the waiter came, he complained; the dog growled in anger at the disrespectful manner it was spoken of, and gave evidence of the fact by seizing the trousers and giving the dreamer a rather savage shake. He struggled to disengage himself, but he could not get rid of his disagreeable assailant, so he urged the waiter to remove him, and he awoke with a laugh, saying, "Well, it's a good thing he did not seize my flesh!"

We believe that the subject of a dream may or may not result from the disturbing influence of particular kinds of food; but the latter will oftentimes, either mediately or immediately, shape and fashion the ideas of the dreamer. For instance, there is something instructive in the dream just related, as the individual had been bitten by a dog when he was a little boy, which seized him by the leg and wounded the calf. So that for many years, to use his own words, 'his politeness to the canine species made him not only throw baits to bribe them into civility, but he carried his deference to such a fastidious degree, that he invariably walked on the opposite side of a street whenever he met a dog, so that he might not be incommoded by it.

Now, it should be remembered that he had exulted at dinner that he had a carnivorous propensity, and this *one* idea had continued to float in his brain, and became the nucleus, so to speak, around which was produced the reminiscence of his old enemy the dog. The dog which had wounded him in his boyhood was of the shepherd breed.

Here is another instance worthy of being recorded:—

A literary man, who was what is called, in ordinary parlance, "a good trencher-man," that is, one who invariably enjoyed his meals, one day had dined at home with his family, and being very hungry, he had dispatched slice after slice of some roast or boiled mutton, declaring that he had never eaten a more delicious, juicy, tender specimen of the far-famed Southdown sheep, and, as a proof of his sincerity, he refused all the *et-ceteras* of a wealthy citizen's dinner meal. After dinner he took his usual short *siesta.*

He had been but a short time asleep when he began to laugh, and that so merrily that it was contagious, and the risible chorus soon awoke him.

Immediately all asked him what he had dreamt about that had so tickled his fancy, and literally "set the table in a roar?" "Well," he replied, "I thought that I was at a public dining-table, not like our market ordinaries, for there were many strangers. The tables were well supplied with fish, meats, and vegetables of all kinds in season, and I myself only partook of mutton.

"But what tickled my fancy in the first instance was, that I overheard the waiters speak of the different visitors, giving each the name of 'the dainty dish' he or she had preferred. There were *Mr. Fish, Mrs. Steak, Miss Stew, Lady Cabbage, Mr. Rice, Miss Duck, Old Squire Bread* (the latter having repeatedly called for the 'staff of life'), and numerous others, distinguished by what they most enjoyed.

"I thought it curious that I had not been mentioned, and probably

I should not have been, had not one of the ladies asked my name. 'Oh,' said the domestic addressed, 'You mean Old Mutton.'"

This audible reply was not considered rude, as he said he had simulated deafness when taking his seat, in order to avoid being annoyed by companions. He thus became the topic of conversation.

"'Old Mutton is a curious, eccentric personage,' observed a sly, funny squinting man of the law, who with a most bewitching lisp proposed 'Old Mutton's health,' 'and let him know our admiration is excited,' said the speaker, 'because he sticks so lovingly to his family connexions, and gives them the preference, as we all have witnessed.'

"All the company arose simultaneously, and turned towards me (whom they deemed oblivious), and then with mock gravity bowed, saying, 'Old Mutton's good health.' Whilst a few, not improved in their manners by what they had drunk, shouted out, 'Here's to jolly Old Mutton.'

"Then I opened my eyes and stared at them, and thus addressed the company: 'Thank ye, my innocent lambs, but you bleat most discordantly.' The surprise that was depicted on every face looked most ridiculous, and I continued, in a strain of badinage, 'Well, you pretty innocent wool-covered creatures, I am very glad you are not foxes, or else "Old Mutton," "Jolly Old Mutton," 'would have had little chance of saving himself from *your* chops!'

"The climax of their absurdity enhanced the fun, as they had actually thought that the waiter had told them my real name, so that the discordant chorus that followed was a second 'Babel confusion.' Take as an example, 'We didn't intend to offend Mr. Mutton.' 'We like mutton.' 'We don't intend to fleece you.' 'Mind your shoulders, Old Mutton.' 'Take care of your legs, Old Mutton:' and so forth.

"These specimens of small wit made the whole party merry, and I joined in the laugh at my own expense. But what rendered the merriment so boisterous, I was standing up, and on the opposite side of the table stood a thin, lantern-jawed, half-starved looking creature, with spindle shanks, on which he had mounted a scarecrow body, vociferating, 'Who cares for Old Fat Mutton?' to which with great gravity I replied, 'Why, surely, you, Mr. Sheepshanks, will not permit our family name to be dishonoured?' The silly fellow protested against our being relations with a most violent stammer and distorted features, and declared 'that he was no connexion of the Muttons, and never cared to be.'" His vehemence and ludicrous appearance called forth peals of laughter, and this awoke the dreamer. And after this account, which is greatly abridged, it may be mentioned that all had taken place during a doze of a few minutes.

Here is an example in which a dream occurring after a supper of tough beef-steak assumed a painful and distressing character:—

"One evening," writes the relater, "we called on a bachelor friend, who insisted that we should stop and take supper with him. The servant brought up a beef-steak which was very hard and tough. This steak, we observed, would have been made tender by beating, which is a quick process of rendering the muscular fibre soft, acting with even greater certainty for this purpose than hanging the meat up for a few days. Our friend smiled, and merely remarked, 'Beating and hanging, then, are useful experiments, in their way?'

"We left him about twelve, and rode home; and it was not long afterwards that we retired to rest. But in less than a quarter of an hour we awoke from a dream, the heart palpitating and the whole body bathed in a cold and clammy perspiration.

"We had dreamt that we were still with our recent companion, and that he was speaking in a most friendly manner, when suddenly he turned round and cursed most vehemently, and then, in a most hurried and irritated way, told us 'to go and drown our carcase, as it was a quicker and less unseemly process than either hanging or cutting the throat.' He then laughed in a very maniacal manner, bent his fist at us in a threatening attitude, saying, 'Why the d——l did you give me the hint to hang myself? If I do it, you'll be hanged, and I'll come as a witness against you.' And then he yelled so frightfully that the dreadful spectacle awoke us.

"Reflecting on this vision of sleep, it occurred to us that, just before leaving, a passing idea floated in our brain, that, from the excitable nature of our friend, care must be taken that he does not go mad. This notion somehow mixed itself up with the conversation about the steak, and with his comment 'that beating and hanging,' &c., and thus was concocted a most painful dream. The proximate cause might have been that the arms were crossed, pressing on the chest, and greatly impeding the respiratory action."

In this essay we have treated of dreams which have been occasioned by food which has disagreed with the digestive organs, or which has exercised a peculiar effect upon the nervous system; of dreams which have been induced by food taken at an improper time, or which have happened when sleep has been indulged in immediately after taking food; and we have recorded certain instances in which not only the dream itself, but even the peculiar train of thought manifested in the dream was occasioned by the special article of food which had, by decoying the palate, disturbed both the stomach and the brain.

